# Female Inheritance of Malarial *lap* Genes Is Essential for Mosquito Transmission

**DOI:** 10.1371/journal.ppat.0030030

**Published:** 2007-03-02

**Authors:** J. Dale Raine, Andrea Ecker, Jacqui Mendoza, Rita Tewari, Rebecca R Stanway, Robert E Sinden

**Affiliations:** Division of Cell and Molecular Biology, Faculty of Natural Sciences, Imperial College London, London, United Kingdom; Washington University School of Medicine, United States of America

## Abstract

Members of the LCCL/lectin adhesive-like protein (LAP) family, a family of six putative secreted proteins with predicted adhesive extracellular domains, have all been detected in the sexual and sporogonic stages of *Plasmodium* and have previously been predicted to play a role in parasite–mosquito interactions and/or immunomodulation. In this study we have investigated the function of PbLAP1, 2, 4, and 6. Through phenotypic analysis of Plasmodium berghei loss-of-function mutants, we have demonstrated that PbLAP2, 4, and 6, as previously shown for PbLAP1, are critical for oocyst maturation and sporozoite formation, and essential for transmission from mosquitoes to mice. Sporozoite formation was rescued by a genetic cross with wild-type parasites, which results in the production of heterokaryotic polyploid ookinetes and oocysts, and ultimately infective *Δpblap* sporozoites, but not if the individual *Δpblap* parasite lines were crossed amongst each other. Genetic crosses with female-deficient *(Δpbs47)* and male-deficient *(Δpbs48/45)* parasites show that the lethal phenotype is only rescued when the wild-type *pblap* gene is inherited from a female gametocyte, thus explaining the failure to rescue in the crosses between different *Δpblap* parasite lines. We conclude that the functions of PbLAPs1, 2, 4, and 6 are critical prior to the expression of the male-derived gene after microgametogenesis, fertilization, and meiosis, possibly in the gametocyte-to-ookinete period of differentiation. The phenotypes detectable by cytological methods in the oocyst some 10 d after the critical period of activity suggests key roles of the LAPs or LAP-dependent processes in the regulation of the cell cycle, possibly in the regulation of cytoplasm-to-nuclear ratio, and, importantly, in the events of cytokinesis at sporozoite formation. This phenotype is not seen in the other dividing forms of the mutant parasite lines in the liver and blood stages.

## Author Summary

Malaria parasites are transmitted between human hosts by female mosquitoes. Following fertilization between male and female gametes in the blood meal, zygotes develop into motile ookinetes that, 24 hours later, cross the mosquito midgut epithelium and encyst on the midgut wall. During this development, parasite numbers fall dramatically and as such, this may be an ideal point at which to disrupt transmission, but first essential parasite targets need to be identified. A protein family implicated in the interactions between parasites and mosquitoes is the LCCL/lectin adhesive-like protein (LAP) family. LAPs are highly expressed in the sexual and ookinete stages, yet when we removed genes encoding each of four LAPs from the genome of a rodent model malaria parasite, a developmental defect was only observed in the oocyst some ten days after the protein was first expressed. These “knockout” parasites did not undergo normal replication and consequently could not be transmitted to mice. Through genetic crosses with parasite mutants producing exclusively either female or male gametes, we demonstrate that parasites can only complete their development successfully if a wild-type *lap* gene is inherited through the female cell. These data throw new light on the regulation of parasite development in the mosquito, suggesting that initial development is maternally controlled, and that the LAPs may be candidates for intervention.

## Introduction

Transmission of the malarial parasite *Plasmodium* from the vertebrate host to the mosquito vector requires rapid sexual development within the mosquito midgut, which is triggered upon ingestion of male and female gametocytes by the mosquito during a blood meal. Gametocyte activation and gametogenesis occur within 15 min, and fertilization between two haploid gametes results in formation of a diploid zygote, usually in the first hour. Zygotes immediately undergo meiosis and differentiate within 24 h into motile, invasive ookinetes. The ookinetes cross the mosquito midgut epithelium and differentiate beneath the basal lamina into oocysts, where circa 11 rounds of endomitosis give rise to up to circa 8,000 haploid nuclei. Sporozoites that finally bud from the oocyst invade the mosquito salivary glands to be transmitted back to a vertebrate host.

Sexual development and midgut invasion represent a major natural population bottleneck in the *Plasmodium* life cycle [1], during which the parasite is critically dependent on intercellular interactions, both between parasite cells (e.g., at fertilization) and between parasite and host. A protein family implicated in these interactions, based on its expression profile and the presence of signal peptides and predicted adhesive extracellular domains, is the *Limulus* clotting factor C, Coch-5b2, and Lgl1 (LCCL)/lectin adhesive-like protein (LAP) family (also referred to as the CCp family; see Table S1).

Six *lap* genes were identified in the *Plasmodium* genome, with *lap2/lap4* and *lap3/lap5* representing putative paralogues [2–8]. LAP1 is conserved across the Apicomplexa and contains a unique mosaic of scavenger receptor cysteine rich (SRCR), polycystine-1, lipoxygenase, alpha toxin/lipoxygenase homology 2 (PLAT/LH2), pentraxin/concanavalin A/glucanase, and LCCL domains. LAP2 and LAP4 contain an LCCL and a predicted lectin domain derived from the fusion of ricin B–like and galactose-binding domains. LAP6 has an LCCL domain and a C-terminal module with homologies to ConA-like lectin/glucanase-, laminin-G-like, and pentraxin domains [4]. The presence of SRCR domains and complex lectin domains in the predicted structures of these proteins has led to the hypotheses that LAP1 may function as an immune modulator [2,6], and that LAP1, 2, 4, and 6 may bind complex polysaccharides that are possibly of mosquito origin [4].

In Plasmodium berghei (pb), LAP1 has been detected in all life stages analyzed (including asexual blood, sexual, and all mosquito stages), LAP2 and LAP4 in gametocytes, ookinetes, and oocysts, and LAP6 in gametocytes, ookinetes, oocysts, and sporozoites ([2–4]; Figure S3; R. Stanway, J. Johnson, J. Yates III, and R. Sinden, unpublished data). The failure to detect PfLAP1, PfLAP2, and PfLAP4 in mosquito stages (ookinetes, oocysts, and sporozoites) by indirect fluorescence antibody assays is particularly intriguing given that PbLAP1 is essential for sporozoite formation in P. berghei [2], and PfLAP1 and PfLAP4 are essential for sporozoite infectivity to the salivary glands in Plasmodium falciparum (pf) [8]. However, as previously noted for the protein MAEBL, negative immunofluorescence data may be indicative only of the absence of a specific epitope, for example, due to conformational changes, proteolytic processing, or interactions with other proteins, and not necessarily the absence of the protein per se (discussed in [4]). Interestingly, in a proteomic analysis of separated male and female gametocytes of *P. berghei,* three members of the family, PbLAP1, 2, and 3, were exclusively detected in female, but not male gametocytes, an expression pattern confirmed in reporter studies [9].

In P. falciparum gametocytes, PfLAP1, 2, and 4 have been detected on the parasite surface, in the parasitophorous vacuole, in vesicles secreted from the parasite into the parasitophorous vacuole, and in the parasite cytoplasm [6,8]. In *P. berghei,* the localization of PbLAP1 is perinuclear in both asexual and sexual blood stages until gametogenesis, after which the protein appears to be relocated to the parasite surface [4]. These observations are all consistent with targeting of the LAPs through the endoplasmic reticulum into vesicles and their subsequent release onto the parasite surface or into the parasitophorous vacuole. Pradel et al. have subsequently demonstrated that surface expression of PfLAP1, 2, and 4 is interdependent, suggesting that the proteins interact functionally [10].

In this study, we have further investigated the functions of PbLAP1, 2, 4, and 6 through phenotypic analysis of P. berghei loss-of-function mutants. We demonstrate that these proteins are critical for oocyst maturation and sporozoite formation. Despite their similarity, the four members of the LAP family characterised in this study do not have mutually redundant functions and are all essential for parasite transmission through the mosquito. Using genetic crosses, we reveal that for sporogony to occur normally, the wild-type (wt) *pblap* genes have to be inherited from the female gametocyte. This leads us to suggest that the observable mutant phenotype in the late oocyst is a functional consequence of the absence of protein function early in parasite development in the mosquito, i.e., at a time when only the female-derived *pblap* genes are being expressed.

## Results

### Targeted Disruption of *pblap2, pblap4,* and *pblap6*

To investigate the function of PbLAP2, 4, and 6, *pblap2, pblap4*, and *pblap6* were independently disrupted via double cross-over homologous recombination and integration of a modified *Toxoplasma gondii dihydrofolate reductase/thymidylate synthase (dhfr/ts)* gene cassette (which confers resistance to pyrimethamine) to create parasites *Δpblap2, Δpblap4,* and *Δpblap6*. The *Δpblap2, Δpblap4,* and *Δpblap6* mutants were verified by diagnostic PCR and Southern blot *(Δpblap2* and *Δpblap4)* or pulsed field gel electrophoresis *(Δpblap6)* analysis (Figure S1). Independent clones were generated and analyzed for each of these parasite lines. A similar approach was previously described to disrupt *pblap1* to create the parasite denoted here as *Δpblap1* [2]. Successful gene deletion was further confirmed by reverse transcriptase (RT)–PCR analysis on *Δpblap* ookinete cDNA, which failed to detect the respective *pblap* mRNA in the corresponding *Δpblap* line, whereas expression of all other *lap* genes was not affected (Figure S1).

### Phenotypic Analysis of *Δpblap* Parasites

Following inoculation of mice with infected blood, the morphologies and production rates of asexual and sexual (male and female) blood stages of *Δpblap1, Δpblap2, Δpblap4,* and *Δpblap6* parasites were indistinguishable from *wt* (unpublished data). All four *Δpblap* lines formed ookinetes (both in vitro and in vivo), which appeared morphologically normal as indicated by observations of Giemsa-stained blood films (unpublished data).

**Figure 1:**
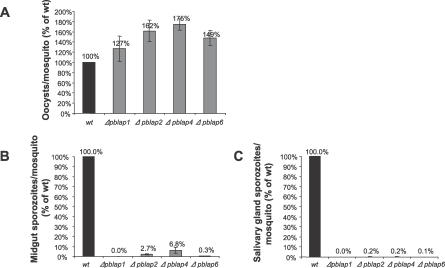
Oocyst and Sporozoite Development of *wt* and *Δpblap* Parasites Graphical summary of oocyst numbers (A), midgut sporozoite numbers (B), and salivary gland sporozoite numbers (C) of *wt* and *Δpblap* parasites. Values of *Δpblap* parasites are given as mean % of *wt* (± standard error of the mean). *Δlap1* as published in [2]. Please refer to Tables S3 and S4 for individual data.

All *Δpblap* parasites were capable of infecting Anopheles stephensi mosquitoes, and on day 10/11 post-infection (p.i.), numbers of oocysts were never less than those observed in *wt*-infected mosquitoes (Figure 1; Table S3). The diameters of *Δpblap1, Δpblap2, and Δpblap6* oocysts were significantly larger than that of *wt* on day 7, and all mutants were larger on days 14 and 21 of infection (Figure S2).

**Figure 2:**
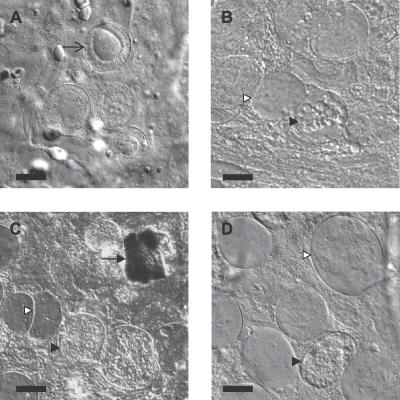
Oocyst Morphology of *wt* and *Δpblap* Parasites Differential interference contrast images of *wt* day 21 p.i. (A), *Δpblap2* day 13 p.i. (B), *Δpblap4* day 18 p.i. (C), and *Δpblap6* day 21 p.i. oocysts (D) in *An. stephensi*. Most *wt* oocysts have undergone sporulation (open arrow). No sporozoite formation is observed in *Δpblap* infections, and oocysts appear either immature/enlarged (open arrowhead) or degenerate/vacuolated (closed arrowhead). Some *Δpblap4* oocysts are melanized (closed arrow). Scale bar = 20 μm.

**Figure 3:**
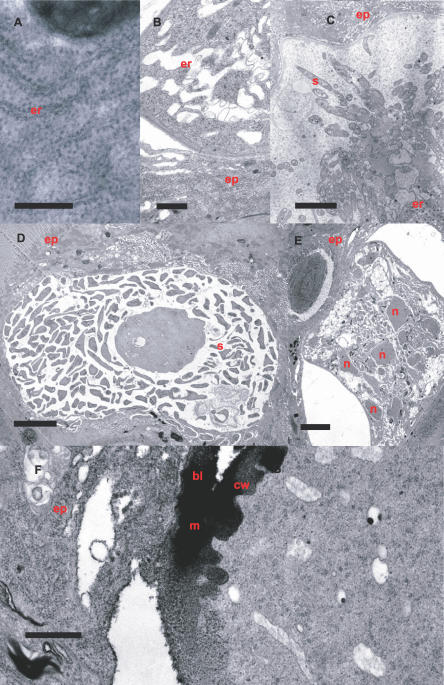
Transmission Electron Micrographs of *wt* and *Δpblap* Oocysts All images taken on day 13 p.i. unless otherwise indicated. Scale bar = 1 μm (A, B, F) or 5 μm (C–E). ep, midgut epithelium. (A) *wt* oocyst showing normal morphology of the endoplasmic reticulum (er). (B) *Δpblap2* oocyst showing extensive expansion of the endoplasmic reticulum (er). (C) *Δpblap1* oocyst (day 27 p.i.) showing extensive expansion of the endoplasmic reticulum (er) and some budding sporozoites (s). (D) *wt* oocyst showing normal morphology following cytokinesis to produce hundreds of daughter sporozoites (s). (E) *Δpblap2* oocyst showing extensive degeneration and few nuclei (some of which are labelled n). (F) Degenerate *Δpblap4* oocyst showing prominent melanization (m) of the extracellular oocyst wall (cw) which appears to spread into the mosquito basal lamina (bl).

Light microscopy revealed the presence of two distinct populations of *Δpblap1, Δpblap2, Δpblap4,* and *Δpblap6* oocysts: those that displayed a phenotype reminiscent of immature *wt* oocysts (i.e., non-sporulated), and those that appeared vacuolated/degenerate compared to *wt* (Figure 2). Transmission electron microscopy analysis of *Δpblap1, Δpblap2,* and *Δpblap4* oocysts further confirmed these findings and revealed that oocysts of these parasites possessed an endoplasmic reticulum that was highly vacuolated compared to that of *wt* parasites (Figure 3). On day 13 p.i., the nuclear organization of *Δpblap1, Δpblap2,* and *Δpblap4* oocysts appeared “immature” as indicated by the presence of few but large nuclei (Figure 3). By comparison, *wt* oocysts of the same age had formed sporozoites, each with their own (haploid) nucleus.

Both light and electron microscopy revealed that some *Δpblap4* oocysts were, unusually, melanized from day 13 p.i. onwards (Figures 2 and 3). Melanization was commonly seen in the oocyst wall and overlying midgut basal lamina, but no melanin deposits were observed in the oocyst cytoplasm, suggesting that in these specimens melanization involved neither parasite plasmalemma nor cytoplasm [11]. Unequivocal melanization of *Δpblap2* oocysts was not observed on day 13 p.i. From day 18–20 p.i. onwards, a variable proportion of *Δpblap2* and *Δpblap4* oocysts had been extensively melanized. Melanization was not observed at any time point (day 10–25 p.i.) in *wt, Δpblap1,* or *Δpblap6* infections.

In contrast to *wt* infections, no midgut sporozoites were observed in *Δpblap2, Δpblap4,* or *Δpblap6* infections on day 10/11 p.i. By day 18 p.i., reduced numbers (typically 0%–12%) of sporozoites were observed in dissected midguts (Figure 1; Table S4). The number of sporozoites in salivary gland preparations from *Δpblap2, Δpblap4,* and *Δpblap6* infections was consistently reduced to <1% of *wt*. No *Δpblap1* salivary gland sporozoites were observed (Table S4), as previously reported [2]. The expression and targeting of the major sporozoite surface protein, circumsporozoite protein, in *Δpblap2, Δpblap4,* and *Δpblap6* midgut sporozoites was indistinguishable from that in *wt* (unpublished data).

The most sensitive method for the detection of infectious salivary gland sporozoites is xenodiagnosis in naïve mice. To test if the observed *Δpblap2, Δpblap4,* and *Δpblap6* sporozoites were infectious to mice, infected mosquitoes were allowed to feed on mice on days 21 and 28 p.i. Blood stage parasites were observed in all mice bitten by *wt*-infected mosquitoes when first screened on day 4/5 post-bite. In contrast, mice bitten by *Δpblap2*-, *Δpblap4*-, and *Δpblap6-*infected mosquitoes remained uninfected until sacrificed on day 14 post-bite (unpublished data).

### Genetic Complementation of *Δpblap* Mutant Phenotypes

**Figure 4:**
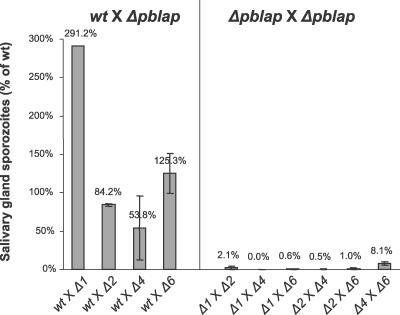
Sporozoite Development in Genetic Crosses Graphical summary of salivary gland sporozoite numbers derived from crosses between *wt* and *Δpblap* and amongst *Δpblap* strains. Values given are mean % of *wt* (± standard error of the mean). In *wt* crosses, diagnostic PCR on blood stage infection resulting from mosquito bite confirmed transmission of the *Δpblap* parasites (not shown). *Δlap1* as published in [4]. Please refer to Table S5 for individual data.

In previous studies, crossing *Δpblap1* gametocytes *(pblap1^−^)* with *wt* gametocytes *(pblap1^+^)* to form heterokaryotic *(pblap1^+^/pblap1^−^)* oocysts rescued the lethal *Δpblap1* phenotype, and produced *Δpblap1* sporozoites that were infectious to mice [4]. Our crosses between *Δpblap1* and *wt* gametocytes produced similar results. Crosses between *Δpblap2, Δpblap4,* and *Δpblap6* and a *wt* clone similarly produced wt numbers of salivary gland sporozoites that were infectious to mice (Figure 4; Table S5). Diagnostic PCR analysis revealed that both *wt* and either *Δpblap2* or *Δpblap4* or *Δpblap6* parasites were present in the blood stage parasites isolated from the infected mice, indicating that *Δpblap* sporozoites (i.e., sporozoites which lack the respective *pblap* gene but which may contain some of the corresponding PbLAP protein carried over from the heterokaryotic oocyst in which they were formed) could be transmitted to mice and that PbLAP2, 4, and 6, like PbLAP1, are not essential for liver or blood stage development (unpublished data).

Given that *pblap1^+^/pblap1* ^−^ *, pblap2^+^/pblap2* ^−^ *, pblap4^+^/pblap4* ^−^, and *pblap6^+^/pblap6* ^−^ heterokaryotic oocysts can produce infectious *Δpblap1, Δpblap2, Δpblap4,* and *pblap6* sporozoites, respectively, we hypothesized that crossing each of the *Δpblap1, Δpblap2, Δpblap4,* and *Δpblap6* mutants with each other to produce heterokaryotic oocysts at two gene loci might rescue the mutant phenotypes described above. However, all potential combinations of crosses between *Δpblap1, Δpblap2, Δpblap4, and Δpblap6* gametocytes failed to rescue sporozoite production to wt levels (Figure 4; Table S5). In these crosses, both the female and male cells have to provide one functional gene copy each, in contrast to the *wt* crosses, where the intact gene copy can be supplied by either cell. Recognizing that (some) *lap* genes are expressed in a sex-specific manner in gametocytes, e.g., PbLAP1, 2, and 3 detected exclusively in female gametocytes [9], we hypothesized that an intact *pblap* gene may only rescue the mutant phenotype when supplied by either a male or a female cell. To test this hypothesis, we performed genetic crosses in vitro with *Δpbs47* and *Δpbs48/45* parasites, which (in vitro) are deficient in forming either female or male functional gametes, respectively ([9,12,13]; C. J. Janse and A. P. Waters, personal communication). Following feeding of the resulting 24-h ookinete culture to mosquitoes, similar numbers of oocysts were observed in mosquitoes infected with *Δpbs47* X *Δpblap* and *Δpbs48/45* X *Δpblap,* but sporozoites were only observed in the *Δpbs48/45* crosses (Table 1). These sporozoites were infectious to C57BL/6 mice. In contrast, mosquitoes infected with *Δpbs47* crosses never transmitted parasites to mice. Diagnostic PCR on genomic DNA prepared from midguts of these mosquitoes demonstrated the presence of the *pblap wt, Δpblap* (and *Δpbs47*) alleles, indicating that crosses between *Δpbs47* and *Δpblap* parasites had occurred (the *Δpblap* parasites thus rescuing the *Δpbs47* parasite to the oocyst stage) (Table 1). Therefore, in all crosses between different *Δpblap* parasites, male gametes (from any *Δpblap* strain) fail to deliver “in time” appropriate expression of their respective *wt* gene to the heterokaryon. Thus, any post-fertilization expression of the male-derived *pblap1, 2, 4,* and *6* genes does not rescue the developmental block at the oocyst stage.

**Table 1:**
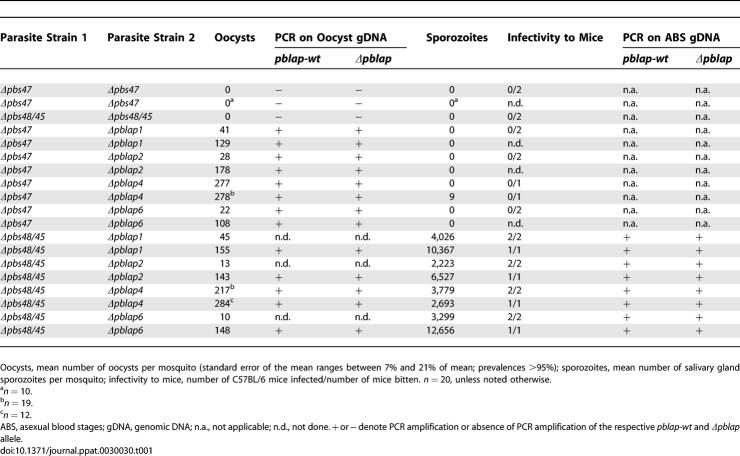
Genetic Crosses between *Δpblap* and *Δpbs47* or *Δpbs48/45*

**Figure 5:**
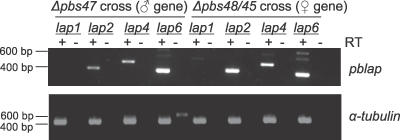
RT-PCR Analysis of *pblap* Expression in Day 10 Oocysts RT-PCR on total RNA isolated from midguts of *An. stephensi* infected with *Δpblap1* × *Δpbs47* (lanes 1 and 2), *Δpblap2* × *Δpbs47* (lanes 3 and 4), *Δpblap4* × *Δpbs47* (lanes 5 and 6), *Δpblap6* × *Δpbs47* (lanes 7 and 8), *Δpblap1* × *Δpbs48/45* (lanes 9 and 10), *Δpblap2* × *Δpbs48/45* (lanes 11 and 12), *Δpblap4* × *Δpbs48/45* (lanes 13 and 14), and *Δpblap6* × *Δpbs48/45* (lane 15 and 16). Top panel, PCR for the respective *pblap* gene as indicated above the panel; bottom panel, control PCR for *a-tubulin;* RT, reverse transcriptase. Fragments of the expected size were amplified for *pblap2* (399 bp), *pblap4* (482 bp), *pblap6* (381 bp), and *α-tubulin* (432 bp). A weak signal for *pblap1* (540 bp) was observed on longer exposures in both crosses. Some genomic DNA contamination was detected for *Δpblap6* × *Δpbs47* (lane 8, bottom panel), but the *pblap6* PCR product from cDNA (lane 7, top panel) can be distinguished from amplification of genomic DNA by the absence of a 310-bp intron.

Recognizing that the *Δpbs47* and *Δpbs48/45* crosses provide a tool to decipher at what point during parasite development the essential PbLAP function occurs (i.e., a time point when a difference in expression of the male and female gene is observed), we undertook RT-PCR analysis of different parasite mosquito stages. RT-PCR analysis on purified ookinetes from ookinete cultures resulting from crosses between either *Δpbs47* or *Δpbs48/45* and *Δpblap* parasites detected strong expression of the *pblap* genes (unpublished data). However, we were unable to prove that this was due to expression of the male gene in a *Δpblap* X *Δpbs47* ookinete, or whether it results from contamination from unfertilized *Δpbs47* females, which also express *lap* genes and inevitably co-purify with ookinetes on anti-Pbs21 magnetic beads. While RT-PCR on young (day 4–5) oocysts is technically challenging and did not give conclusive results, RT-PCR on day 10 oocysts, in which the mutant phenotype becomes apparent, clearly detected expression of the male nucleus-derived *pblap* genes (Figure 5).

Finally, to test whether genes derived from the male nucleus are accessible to the transcription and translation machinery in the zygote/ookinete, we crossed *Δpbs47* and *Δpbs48/45* with a parasite line disrupted in a member of the micronemal membrane attack complex/perforin domain containing *Plasmodium* perforin-like protein (PPLP) family. This parasite is non-infective to mosquitoes due to a loss of midgut-invasion capacity [14]. This mutant phenotype was rescued equally by a cross with either *Δpbs47* or *Δpbs48/45* (unpublished data).

We therefore conclude that despite a late observable mutant phenotype some 10 d after mosquito infection, essential PbLAP functions occur early in the parasite development in the mosquito, i.e., either in the female gametocyte/gamete or in the zygote/ookinete following fertilization, but before expression of the male gene copy.

## Discussion

Our studies have demonstrated that inheritance of *pblap1, 2, 4,* and *6* from the female gametoctyte is essential for parasite development, indicating that the critical functions of the LAPs cannot be provided by the male gene copy. While the mutant phenotype in the *Δpblap* parasites becomes apparent only around day 10 of oocyst development, we demonstrate by RT-PCR analysis of different genetic crosses that both the male and female gene copy are expressed at this time point. As expression of the male gene by day 10 (and possibly day 4; unpublished data) of oocyst development is too late to rescue the defect in oocyst maturation and sporulation, we conclude that the observed mutant phenotype must be a consequence of the absence of protein function earlier in development. More precisely, the critical function must occur at a time point when the male and female gene are expressed in a differential manner. This has so far only been described at the gametocyte stage. Strikingly, it was shown that (at least) PbLAP1, PbLAP2, and PbLAP3 were exclusively detected with high abundance in female gametocytes, and this sex-specific expression was confirmed by reporter studies [9].

Unfortunately, not much is known about the pattern of gene expression from the male and female genome post-fertilization. We attempted to analyse *pblap* gene expression in ookinetes derived from genetic crosses with male- and female-deficient lines, but were not able to obtain sufficiently pure preparations. Therefore, we could not determine at what time point post-fertilization the male *pblap* genes are first transcribed and thus whether LAP function is critical pre-fertilization in the female gametocyte/gamete, or post-fertilization during early zygote/ookinete development before the male-derived genes are expressed appropriately. However, a PPLP knockout was complemented with a male-derived *pplp* gene within the first 24 h post-fertilization (i.e., before midgut invasion), indicating that (some) male genes are expressed before this time point. Clearly, further studies are required to understand the pattern of *pblap* gene regulation in the sexual and sporogonic stages of *Plasmodium* development. Specifically, introducing *pblap* promoter–reporter constructs into male- and female-deficient lines will help narrow down the critical time point of activity.

An early essential function of LAPs at the gametocyte/ookinete stage would be consistent with the high protein expression detected for PbLAP1–5 in these stages [4]. We note that while published studies have failed to detect the expression of PbLAP6 before the sporozoite stage, we have subsequently detected the mRNA in gametocytes, ookinetes, and oocysts by RT-PCR (Figure S3) and the protein in ookinetes by multidimensional protein identification technology analysis (R. Stanway, J. Johnson, J. Yates III, and R. Sinden, unpublished data). Gene expression in the female gametocyte is unusual in that a number of mRNAs are translationally repressed until gametocyte activation, possibly to regulate gene expression during meiosis in the zygote [3,13]. Based on the detection of abundant LAP protein in gametocytes of both P. berghei and P. faciparum in a number of studies [3–10], we consider it unlikely that the majority of *lap* transcripts are translationally repressed. We therefore note with interest that the mRNAs for *pblap4, 5,* and *6,* but not *pblap1,* were destabilised in a knockout parasite incapable of translational repression [13].

The precise cellular function of the LAPs remains enigmatic. The morphological characterisation of *Δpblap1, Δpblap2, Δpblap4,* and *Δpblap6* clones in P. berghei has revealed the same mutant phenotype in the oocyst. This is consistent with the suggestion that the LAPs work either in a functional cascade or as a complex [10]. Our data suggests that in these mutants, regulation of the cell cycle is lost. The increase in oocyst size, the large proportion of oocysts displaying a vacuolated/degenerate phenotype, the presence of large but few nuclei, and the reduction *(Δpblap2, Δpblap4,* and *Δpblap6)* or complete absence *(Δpblap1)* of salivary gland–associated sporozoites all suggest that these mutants are unable to regulate the classic nuclear-to-cytoplasmic relationship that reportedly controls cytokinesis. Although few *Δpblap2, Δpblap4,* and *Δpblap6* sporozoites were detected in salivary gland preparations, in this study none could be transmitted to mice via mosquito bite, suggesting either that the number of sporozoites inoculated is too low to establish an infection, or possibly that these sporozoites are non-infectious to mice. Loss of function of the *Δpblap* parasites is rescued in *pblap* ^+^ */pblap* ^−^ heterokaryons ([4]; this study), and all resulting haploid *Δpblap* sporozoites that bud from the polyploid sporoblast are able to complete the exo-erythrocytic, erythrocytic, and sexual phases of the life cycle. This raises the fascinating question as to why putative cell cycle defects are only observed in the oocyst and not in the exo-erythocytic or erythrocytic schizonts.

The probable surface location of the LAP proteins on the surface of gametocytes, gametes, zygotes [4,6,8], and sporozoites [4] supports earlier suggestions that these proteins may be involved in parasite–parasite or parasite–host interactions [2,4]. It will be interesting to determine whether this location makes the molecules vulnerable to immunological or chemotherapeutic attack. We present evidence here that *Δpblap2* and particularly *Δpblap4* parasites may be more susceptible to immune attack by the mosquito melanization response at the oocyst stage, though whether this is a direct causal interaction or the simple consequence of parasite death cannot be determined. The latter interpretation would be consistent with the vacuolated appearance of the mutant oocysts. The former hypothesis might suggest that LAP2 and LAP4 may be integrated into the oocyst wall, where they may have a protective function suppressing the mosquito's melanotic response. An immunomodulatory role has also been suggested for LAP1 (based on the prediction of scavenger receptor domains in the protein structure [2,6]).

A study in P. falciparum [8] reports phenotypes of *Δpflap1* and *Δpflap4* different from those of *Δpblap1* and *Δpblap4* (this study, [2]) in that oocyst numbers and morphology in infections in Anopheles freeborni were similar to *wt*. Furthermore, the same study also reports that both *Δpflap1* and *Δpflap4* form sporozoites normally, but that they do not reach the salivary glands during the observation period. However, given that the sample size in their study (between five and 20 infected mosquitoes per experiment) was below that needed to make statistically significant determinations [15], and given the absence of quantitative data, it is impossible to conclude at this stage that the described differences are real. Our data clearly show that it is mainly the formation of sporozoites that is disrupted, although we cannot exclude a role in the transition of the few sporozoites formed from the midgut to the salivary gland. We would also draw attention to the dynamic relationship that exists between parasite and mosquito being dependent upon both parasite strains and mosquito species. Therefore, we do not discount that the mosquito species in which the experiments were conducted *(An. stephensi* versus *An. freeborni)* may also be a factor with regard to the observations made. Taken together, we cautiously conclude the phenotypes may be similar in the two parasite species.

In summary, we demonstrate that expression of the female nucleus-derived *pblap1, 2, 4,* and *6* genes is essential for parasite development in the mosquito, i.e., that the function of PbLAP1, 2, 4, and 6 is critical *prior to* the expression of protein from the male-derived gene copy during sporogony, possibly in the gametocyte-to-ookinete period of differentiation. The absence of PbLAP gene function at this critical period of activity ultimately results in lethality some 10 d later, at sporulation, which represents the endpoint of several complex developmental cascades [16]. The mutant phenotype detectable by cytological methods suggests a key role of the LAP proteins or LAP-dependent processes in the regulation of the cell cycle and, critically, in the events of cytokinesis. Importantly, this phenotype is not seen in the other dividing forms of the parasite in the liver and blood stages.

## Materials and Methods

Parasite maintenance, ookinete cultures, mosquito infections, mosquito bite-back experiments, diagnostic PCR, pulsed field gel electrophoresis, Southern blotting, and transmission electron microscopy were carried out as previously described [17–21].

Locus names of *pblap* and *pflap* genes are listed in Table S1.

### Knock-out parasite construction.

Generation of constructs for targeted disruption of *pblap2, pblap4,* and *pblap6* by double homologous recombination were carried out as previously described [17,22]. Briefly, upstream homology regions of 611 bp *(pblap2* and *pblap4)* or 476 bp *(pblap6)* were PCR amplified from P. berghei ANKA clone 2.34 genomic DNA using primers DR0006F-*pblap2*/ApaI and DR0006R-*pblap2*/HindIII, DR0008F-*pblap4*/ApaI and DR0008R-*pblap4*/HindIII, or AE25a-*pblap6*/ApaI and AE25b*-pblap6*/HindIII, and cloned into pBS-DHFR via ApaI and HindIII. Downstream homology regions of 632 bp *(pblap2),* 594 bp *(pblap4),* or 528 bp *(pblap6)* were PCR-amplified using primers DR0007F-*pblap2*/EcoRV and DR0007R-*pblap2*/BamHI, DR0009F-*pblap4*/EcoRV and DR0009R-*pblap4*/BamHI, or AE25c-*pblap6*/EcoRI and AE25d-*pblap6*/BamHI, and cloned into the plasmids with the respective upstream homology region via EcoRV and BamHI *(pblap2* and *pblap4)* or EcoRI and BamHI *(pblap6)*. The targeting cassette was released by ApaI and BamHI digestion. Parasite transfection using the Human T Cell Nucleofector Kit (amaxa, http://www.amaxa.com) and selection by pyrimethamine and dilution cloning were carried out as previously described [23,24]. Integration of the targeting cassette into the genome leads to replacement of the central 2,950 bp of the coding region of *pblap2* (4,848 bp), 4,454 bp of the coding region of *pblap4* (5,151 bp) and 4,525 bp of the coding region of *pblap6* (4,732 bp) with the drug-selectable marker *T. gondii dhfr/ts*.

### Genetic crosses.

Genetic crosses between gametocytes of different clones were carried out either by mixing equal numbers of gametocytes in normal mouse blood and feeding to mosquitoes via membrane feeding as described previously [4], or by allowing mosquitoes to feed directly on mice infected with different parasite combinations. No difference was seen between these two methods. For the crosses with *Δpbs47* or *Δpbs48/45,* mice were infected with the different parasite combinations, and ookinetes were cultured in vitro and fed to mosquitoes via membrane feeding at a concentration of 800 ookinetes/μl in normal mouse blood. Genomic DNA from oocysts (on midguts) was prepared using the Wizard genomic DNA Purification Kit (Promega, http://www.promega.com) following the protocol for “mouse tail”. The *pblap2, pblap4,* and *pblap6 wt* alleles were amplified using primers lapX-KO and lapX-WT. The *Δpblap2, Δpblap4,* and *Δpblap6* alleles were amplified using primers lapX-KO and 248. Diagnostic PCR for *pblap1* was as previously described [4].

### RT-PCR.

Total RNA was isolated using TRIzol (Invitrogen) according to the manufacturer's instructions. Contaminant genomic DNA was removed by treatment with TURBO DNA-*free* (Ambion, http://www.ambion.com) and RNA was cleaned up using the RNeasy Mini Kit (Qiagen, http://www1.qiagen.com). Reverse transcription was performed on 1 μg of RNA using the TaqMan Reverse Transcription Reagents with a mixture of Oligo-dT primers and Random Hexamers (Applied Biosystems, http://www.appliedbiosystems.com), and the resulting cDNA was used in diagnostic PCR reactions. Primers SRCR3 and SRCR5 amplify a 540-bp fragment of *pblap1,* primers 2RT-F and LAP2WT a 399-bp fragment of *pblap2,* primers 4RT-F and LAP4WT a 482-bp fragment of *pblap4,* primers 6RT-F and 6RT-R a 381-bp fragment of *pblap6,* primers TubF and TubR a 432-bp fragment of the *α-tubulin* gene, and primers p28F and p28R a 642-bp fragment of *pbs21*.

### Primer sequences.

For primer sequences, please refer to Table S2.

## Supporting Information

Figure S1 Generation of *Δpblap2, Δpblap4,* and *Δpblap6* Parasite Lines (A–C) Schematic diagrams of *pblap2, pblap4,* and *pblap6* gene disruption strategies. Sites for homologous recombination (approximately 450–600 bp) are indicated by shading with diagonal lines; *pbdhfr/ts 5′UTR* (2.3 kb), vertical lines; *tgdhfr/ts* selectable marker (1.8 kb), black; *pbdhfr/ts 3′UTR* (483 bp), horizontal lines. Arrows indicate translational start site (ATG) and stars represent the predicted stop codon. Restriction sites utilized in the Southern blot analysis are indicated. Block arrows show position of primers used in diagnostic PCRs (shown in [C] only).
(D) Southern blot analysis of ClaI/XbaI-digested (left) or BglII/XbaI-digested (right) genomic DNA from *wt* and *Δpblap2* (left) or *Δpblap4* (right), hybridized with 5′ probes.
(E) Ethidium bromide–stained pulsed field gel (lanes 1 and 3) with separated chromosomes from *wt* and *Δpblap6* and Southern blot analysis (lanes 2 and 4) hybridized with a *pbdhfr 3′UTR* probe. The open arrowhead indicates the position of the endogenous *pbdhfr 3′UTR* on Chromosome 7, the closed arrowhead the position of the integrated targeting construct on Chromosome 4 (as predicted [25]) of *Δpblap6*. Chr., chromosome.
(F) Diagnostic PCR on genomic DNA from *Δpblap* clones and control *wt* parasites. Primers lapX-WT and lapX-KO amplify the *wt* allele (1,161 bp *lap2,* 1,438 bp *lap4,* 840 bp *lap6*), primers lapX-KO and 248 amplify the disrupted allele (1,182 bp *lap2,* 1,172 bp *lap4,* 867 bp *lap6*).
(G) RT-PCR analysis of *pblap* expression on total RNA isolated from purified in vitro cultivated *Δpblap* ookinetes demonstrates absence of transcript in the respective knock-out. Expected fragments sizes as in Figure 5; *p28* (642 bp). RT, reverse transcriptase.
(144 KB PDF)

Figure S2 Mean Diameter of *wt* and *Δpblap* Oocysts in *An. stephensi*
*Δpblap1* (black cross), *Δpblap2* (black triangle), *Δpblap4* (black square), and *Δpblap6* (black diamond) form oocysts with statistically significantly larger diameter (*z*-test, *p* < 0.001 for all parasites and time points, except *Δlap4* at 7 d) than *wt* (open square and open triangle, representing two independent repeats). Each time point shows an average of 44–215 oocysts. Standard error of the mean were negligible. *Δpblap1* as observed in [2].
(44 KB PDF)

Figure S3 RT-PCR Analysis of *pblap* Expression during Mosquito Development RT-PCR on total RNA isolated from *wt* gametocytes, purified in vitro cultivated *wt* ookinetes, and midguts of *An. stephensi* infected with *wt* and dissected on day 5, 10, and 20 of infection. Fragments sizes as in Figure S1.
(70 KB PDF)

Table S1 Locus Names for *P. berghei lap* Genes and Their Respective P. falciparum Orthologues (17 KB XLS)

Table S2 Primer Sequences (20 KB XLS)

Table S3 Oocyst Development in *Δpblap1, Δpblap2, Δpblap4,* and *Δpblap6*
(24 KB XLS)

Table S4 Sporozoite Development in *Δpblap1, Δpblap2, Δpblap4,* and *Δpblap6*
(21 KB XLS)

Table S5 Genetic Crosses between *wt* and *Δpblap* and amongst *Δpblap* Strains (22 KB XLS)

### Accession Numbers

The PlasmoDB (http://www.plasmodb.org) or National Center for Biotechnology Information (http://www.ncbi.nlm.nih.gov) accession numbers for the genes discussed in this paper are *pblap1* (PB000977.02.0), *pblap2* (PB000652.01.0), *pblap4* (PB000504.02.0), and *pblap6* (PB000955.03.0).

## Abbreviations

*dhfr/ts*: *dihydrofolate reductase/thymidilate synthase*
LAP: LCCL/lectin adhesive-like protein
LCCL: Limulus clotting factor C, Coch-5b2, and Lgl1
pb: Plasmodium berghei
pf: Plasmodium falciparum
p.i.: post-infection
RT-PCR: reverse transcriptase PCR
SRCR: scavenger receptor cysteine rich
wt: wild-type
