# Supplementary material for: Female Inheritance of Malarial lap Genes Is Essential for Mosquito Transmission
Source: PLoS Pathog. 2007 Mar 2;3(3):e30. doi: 10.1371/journal.ppat.0030030 (PMC1808070; doi:10.1371/journal.ppat.0030030)

Supplementary Figure 3

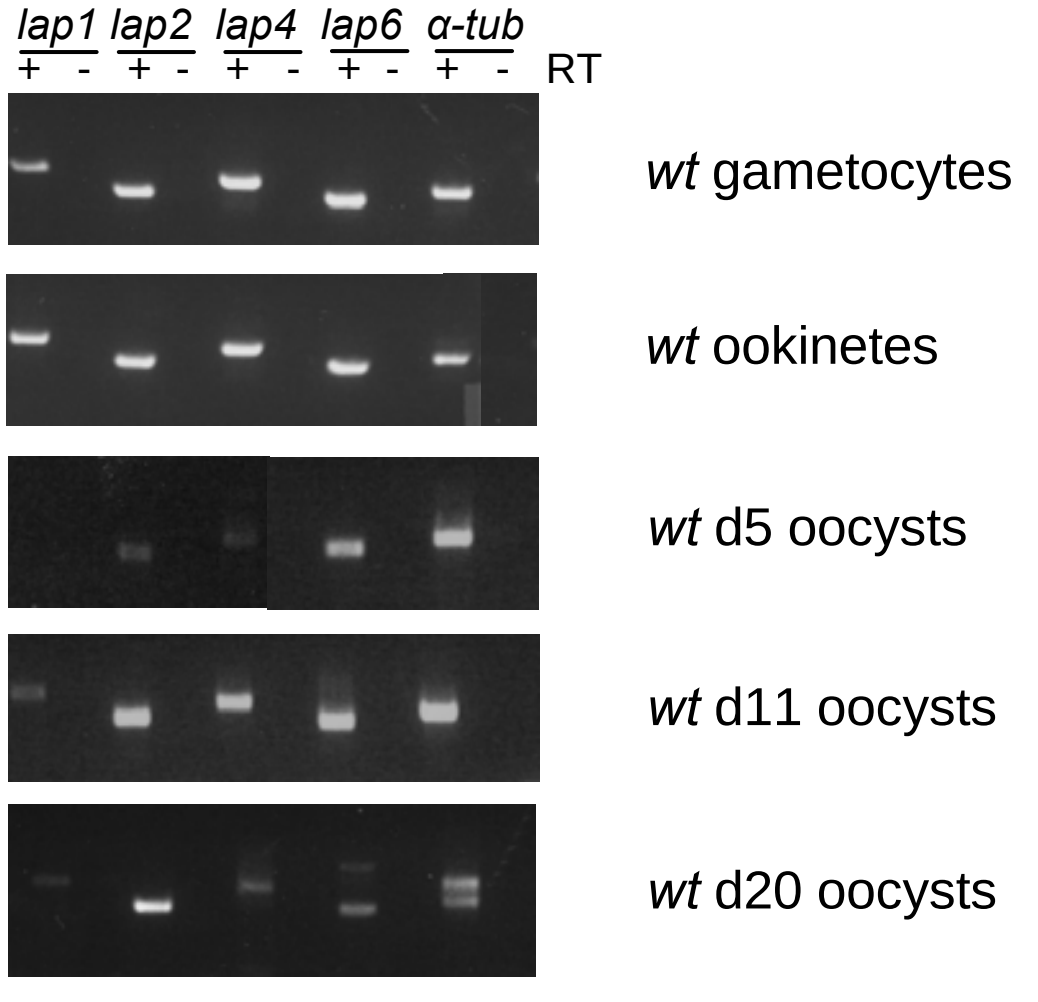

Supplement: Supplementary file 3 [file ppat.0030030.sg003.pdf]
